# The Immune Response to SARS-CoV-2 Vaccine in a Cohort of Family Pediatricians from Southern Italy

**DOI:** 10.3390/cells12111447

**Published:** 2023-05-23

**Authors:** Paolo Cortese, Felice Amato, Antonio Davino, Raffaella De Franchis, Speranza Esposito, Immacolata Zollo, Marina Di Domenico, Egle Solito, Federica Zarrilli, Laura Gentile, Gustavo Cernera, Giuseppe Castaldo

**Affiliations:** 1 Federazione Italiana Medici Pediatri (FIMP), 80142 Naples, Italy; pacortese@libero.it (P.C.); davinoant@gmail.com (A.D.); raffaelladefranchis@gmail.com (R.D.F.); 2 Dipartimento di Medicina Molecolare e Biotecnologie Mediche, Università di Napoli Federico II, 80131 Naples, Italy; felice.amato@unina.it (F.A.); immacolata.zollo@unina.it (I.Z.); federica.zarrilli@unina.it (F.Z.); giuseppe.castaldo@unina.it (G.C.); 3 CEINGE Biotecnologie Avanzate Franco Salvatore, Scarl, 80131 Naples, Italy; espositosp@ceinge.unina.it (S.E.); gentilela@ceinge.unina.it (L.G.); 4 Dipartimento di Medicina di Precisione, Università degli Studi della Campania Luigi Vanvitelli, 80138 Naples, Italy; marina.didomenico@unicampania.it; 5 Centre for Translational Medicine and Therapeutics William Harvey Research Institute, Queen Mary Univesity, London E1 4NS, UK; e.solito@qmul.ac.uk

**Keywords:** COVID-19, SARS-CoV2 antibodies, TNF-α, IFN-γ, T-cell

## Abstract

In Italy, from January 2021, the Ministry of Health indicated a vaccination plan against COVID for frail patients and physicians based on a three-dose scheme. However, conflicting results have been reported on which biomarkers permit immunization assessment. We used several laboratory approaches (i.e., antibodies serum levels, flow cytometry analysis, and cytokines release by stimulated cells) to investigate the immune response in a cohort of 53 family pediatricians (FPs) at different times after the vaccine. We observed that the BNT162b2-mRNA vaccine induced a significant increase of specific antibodies after the third (booster) dose; however, the antibody titer was not predictive of the risk of developing the infection in the six months following the booster dose. The antigen stimulation of PBMC cells from subjects vaccinated with the third booster jab induced the increase of the activated T cells (i.e., CD4^+^ CD154^+^); the frequency of CD4^+^ CD154^+^ TNF-α^+^ cells, as well as the TNF-α secretion, was not modified, while we observed a trend of increase of IFN-γ secretion. Interestingly, the level of CD8^+^ IFN-γ^+^ (independently from antibody titer) was significantly increased after the third dose and predicts the risk of developing the infection in the six months following the booster jab. Such results may impact also other virus vaccinations.

## 1. Introduction

The severe acute respiratory syndrome coronavirus 2 (SARS-CoV-2) started to spread in Wuhan (China) in the last weeks of 2019, and in March 2020, it became a pandemic, causing about 7 million deaths so far at the end of 2022 (World Health Organization (WHO)). The clinical manifestation of the new syndrome was heterogeneous, from asymptomatic infection to critical and systemic disease [1]. Scientists worked hard to develop effective vaccines against the virus [2] that started to be used since December 2020, even if with great differences in vaccinal strategies among countries. In Italy, risk groups, among which were frail patients and physicians, performed a 3-dose scheme that started in January 2021, followed by a second administration after 21 days (February 2021) and a third booster dose after 8 months.

Since 2020, mutations in the SARS-CoV-2 genome caused the appearance of variants of interest (VOIs) that rapidly spread all over the world [3]. Some of such variants spread more rapidly, including B1.1.1.7 (alpha infection was detected until mid-2021), B.1.617.2 (delta spreading lasted until early 2022), and finally, the variant B.1.1.529 (omicron and its subvariants detected up to date), as derived from data of the Health Ministry in Italy (Italian National Institute of Health). The different variants overlapped and succeeded each other with their specific characteristics, such as the high pathogenicity for alpha and delta variants or the high infectivity and milder symptoms for omicron [4,5].

The generation of an effective vaccine occurred within less than a year from the beginning of the pandemic. The vaccines designed toward the spike protein of the Wuhan strain, although not protecting against re-infection, retained a protective role against the severe form of the disease that required intensive care [6]. Currently, more than 180 vaccine candidates are in clinical trials; thus, the availability of effective biomarkers of immunization is of paramount relevance.

The first studies after the vaccine introduction considered the serum antibody level and antibody kinetics as biomarkers of immunization [7], but it rapidly became clear that the antibody titer quickly declined, thus it could not be considered an effective indicator of immunization [8,9]. Recent reports consider the SARS-CoV-2 specific memory T cells crucial to assess long-term immune protection against COVID-19 [10,11]. Different approaches have been used so far to assess the T-cell response to the vaccine, including the analysis of specific populations of T cells producing tumor necrosis factor (TNF)-α or interferon (IFN)-γ [12,13], or the release of IFN-γ (i.e., the IFN-γ release assay—IGRA test) by T cells from vaccinated subjects stimulated with the spike protein [14,15]. It is now clear that the mRNA COVID-19 vaccines, BNT162b2 and mRNA-1273, induce a spike-specific CD8^+^ T-cell response in most individuals. The T cells, indeed, might exert a critical role in providing protective immunity through the T-cell help to B cells to produce affinity matured antibodies and memory B cells. However, most studies described the lack of correlation between the humoral and cellular immune response [16,17].

For this reason, we optimized several laboratory approaches to evaluate the T-cell response and compared T-cell-derived biomarkers to antibody levels in a cohort of family pediatricians (FPs) from Southern Italy undergoing vaccine immunization.

## 2. Materials and Methods

### 2.1. Study Participants

The study was performed according to the principles of the Declaration of Helsinki and was approved by the Ethical Committee of the University of Naples Federico II (approval number 76.21). Informed consent was obtained from all participants. Peripheral blood samples were collected from a group of 53 family pediatricians (FPs) from Naples (Southern Italy), which included 36 females and 17 males (Table 1) who had received the BNT162b2-mRNA vaccine against SARS-CoV-2.

Demographic data were collected at the time of enrollment. All the subjects were free from chronic diseases that could potentially impair their immunological functions. All subjects experienced three doses of the BNT162b2 vaccine against SARS-CoV-2; the samples were collected at three different times (Table 1), namely, T1: one month after second dose; T2: eight months after second dose; T3 one month after third dose. The evaluations were performed at different times, as indicated in Table 1, between January 2021 and May 2022. Our study started with the evaluation of serum levels of IgG targeting the Receptor-Binding Domain (RBD). When it became clear that this parameter was not predictive of the risk of developing the infection, we proceeded to also analyze the T-cell response starting 8 months after the second dose, i.e., T2 time.

All FPs were swabbed every 15 days for the whole period of our study, or immediately at the presence of symptoms.

During this period, different variants of interest (VOIs) of SARS-CoV-2 circulated in Southern Italy (Table 2).

### 2.2. Evaluation of SARS-CoV-2-Specific IgG

Chemiluminescent microparticle assay (CMIA) was used to quantify Anti-Spike RBD-specific IgG serum levels, according to the manufacturer’s instructions (SARS-CoV-2 IgG 6R86 Abbott, Chicago, IL, USA). The antibody reactivity was expressed in AU/mL.

### 2.3. Sample Collection and Storage

Venous blood was collected in EDTA tubes and processed within 3 h. Peripheral blood mononuclear cells (PBMCs) were isolated by density-gradient on lymphocyte separation media (Biowest, Riverside, MO, USA) using Lymphosep tubes (163,290 Grenier Bio-one, Kremsmünster, Austria), according to the manufacturer’s instruction. In brief, blood was diluted with PBS at a 1:1 ratio and centrifuged for 15 min at 1000 rpm. The mononuclear cell layer was isolated, and cells were washed with PBS. Then, PBMCs were cryopreserved in heat-inactivated fetal bovine serum (Gibco, Grand Island, NE, USA) containing 10% DMSO (Sigma, Livonia, MI, USA) and frozen at −80 °C before being stored in liquid nitrogen until use in the assays.

### 2.4. Antigen Stimulation

PBMCs were thawed and rested overnight in complete RPMI media with 5% human AB serum (Sigma). On the day of stimulation, to characterize the specific T-cell response by flow cytometry, PBMCs were plated in a 96-U well plate at 1 × 10^6^ cells per well (100 µL) in a complete medium in the presence of 1 μg/mL^−1^ monoclonal antibody CD28 and CD49d (BD, Franklin Lakes, New Jersey, USA), and for each family pediatrician, the cells were stimulated with peptide pools and treated with the same volume of mock solution (sterile water/10%DMSO) as a response to the control condition [18,19]. SARS-CoV-2 peptide pools of the protein S at 1 ug/mL were used for the stimulation of cultured PBMCs. The peptide pool consists of 15-mer sequences with 11 amino acids overlapping, covering the N-terminal S1 domain of the surface glycoprotein of SARS-CoV-2. The PepTivator SARS-CoV-2 Prot_S1 (Miltenyi Biotec, Bergisch Gladbach, Germany) contains the aa sequence 1–692 of the Wuhan strain spike protein. In addition, CytoStim (Miltenyi Biotec) was used to stimulate PBMCs as positive controls. After 2 h of incubation at 37 °C (5% CO_2_), Brefeldin A (Miltenyi Biotec) was added to cell cultures to inhibit cytokine secretion. Following an incubation of 14 h, cells were harvested and stained as described below. To account for the specific activation in the same individual, the response to the control condition was subtracted from the stimulated sample.

### 2.5. Flow Cytometry

The staining of the cells was performed using the following anti-human antibodies: CD4 FITC, IFN-γ PE, TNF-α Pe-Vio770, CD3 APC, CD154 APC-Vio770, CD14 and CD20 VioBlue, CD8 VioGreen. This flow antibody panel and all reagents used were purchased from Miltenyi Biotec [20,21]. In brief, after the stimulation, the cells were fixed with Inside Fix containing 3.7% formaldehyde (Miltenyi Biotec) for 20 min at room temperature (RT), then centrifugated, washed, and permeabilized with Inside Perm (Miltenyi Biotec) and stained for lineage and activation markers, according to manufacturers’ instructions. The stained cells were acquired with a MACSQuant cytometer (Miltenyi Biotech, Bergisch Gladbach, Germany) and analyzed with MACSQuantify software (Miltenyi Biotec). Dead cells, doublets, and debris were excluded from the analysis by side/forward scatter gating, and CD14^+^ and CD20^+^ cells were excluded, as well. After gating on CD3, as well as CD4 and CD8, activation markers and cytokines expression were assessed, e.g., CD154 and TNF-α for CD4^+^ T cells and IFN-γ for CD8^+^ T cells.

### 2.6. Cytokines Release Assay

Cytokines release was employed to determine the magnitude of the SARS-CoV-2-specific T-cell response. Supernatants from 1 × 10^6^ PBMC, stimulated with PepTivator SARS-CoV-2 Prot_S1 (Miltenyi Biotec) for 16 h, were analyzed by ELLA, with microfluidic multiplex cartridges measuring IFN-γ and TNF-α values, following the manufacturer’s instructions (ProteinSimple, San Jose, CA, USA). The cells were stimulated, as already reported for the flow cytometry analysis without the addition of Brefeldin A. The Stimulation Index (SI) was calculated by dividing the cytokine concentration produced by each subject in response to the peptide pools by the cytokine concentration secreted by the same individual in response to the control condition. The detection limit of these assays was 0.17 pg/mL for IFN-γ and 0.3 pg/mL for TNF-α.

### 2.7. Statistical Analyses

Graphics and statistical analyses were performed with Graph Pad Prism 8 Software (GraphPad Software, San Diego, CA, USA). Quantitative variables were represented as mean ± standard deviation (SD). To assess statistically significant differences in variables following a non-normal distribution, the Wilcoxon test for paired samples was used, while the Mann–Whitney test was used for unpaired samples (between groups A and B). Differences were considered statistically significant at *p* < 0.05, and individual *p* values are indicated in the text and/or figure legends. The Spearman rank test was used for correlation analyses between CD8^+^ IFN-γ^+^ cells and antibody titers.

## 3. Results

### 3.1. Study Group and Analysis of Humoral Response

More appropriate knowledge of the host immune responses to the SARS-CoV-2 vaccine is crucial to assess their effects. Thus, we started our experiments with the evaluation of the humoral immune response.

**Figure 1:**
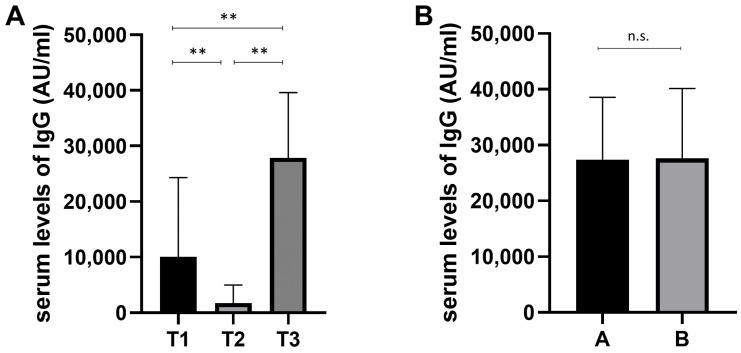
(**A**) Serum levels of IgG (AU/mL) targeting the Receptor-Binding Domain (RBD) in 53 family pediatricians at different times (see Table 1); ** *p* < 0.001. (**B**) Comparison of IgG levels among FPs who developed acute SARS-CoV-2 infection (A, n = 20) and FPs who did not develop acute SARS-CoV-2 infection (B, n = 33) during the 6 months following the third dose of vaccine; n.s. not significant. Means and SD are shown. Statistical analysis was performed as reported in the methods.

The blood samples from vaccinated FPs were collected, as described in Table 1. Data in Figure 1 show the humoral immune response after the corresponding sampling (T1, T2, and T3), assessed by determining the serum levels of IgG (AU/mL) targeting the Receptor-Binding Domain (RBD). As shown in Figure 1A, at T3, the level of serum IgG was significantly higher as compared to T1 and T2, while at T2, the level of serum IgG was significantly lower as compared to T1 and T3. These data show that the booster dose strengthens the humoral response.

During the 6 months after the third dose, 20/53 (37.7%) subjects developed SARS-CoV-2 acute infection (group A), while 33/53 (62.3%) did not develop the infection (group B). Serum IgG levels between the two groups were not significantly different (Figure 1B), thus leading to the conclusion that the serum IgG levels cannot be considered a valid marker in order to evaluate the risk of infection.

Moreover, in our cohort, we found no differences in serum levels of IgG with respect to gender and age.

### 3.2. T-Cell Response

Since the antibody titer alone was not an effective predictor of immunization, we started to evaluate the T-cell response at time T2.

PBMC cells derived by FPs at T2 and T3 were stimulated with the peptides, as reported in the Material and Methods section, stained, and processed by flow cytometry.

**Figure 2:**
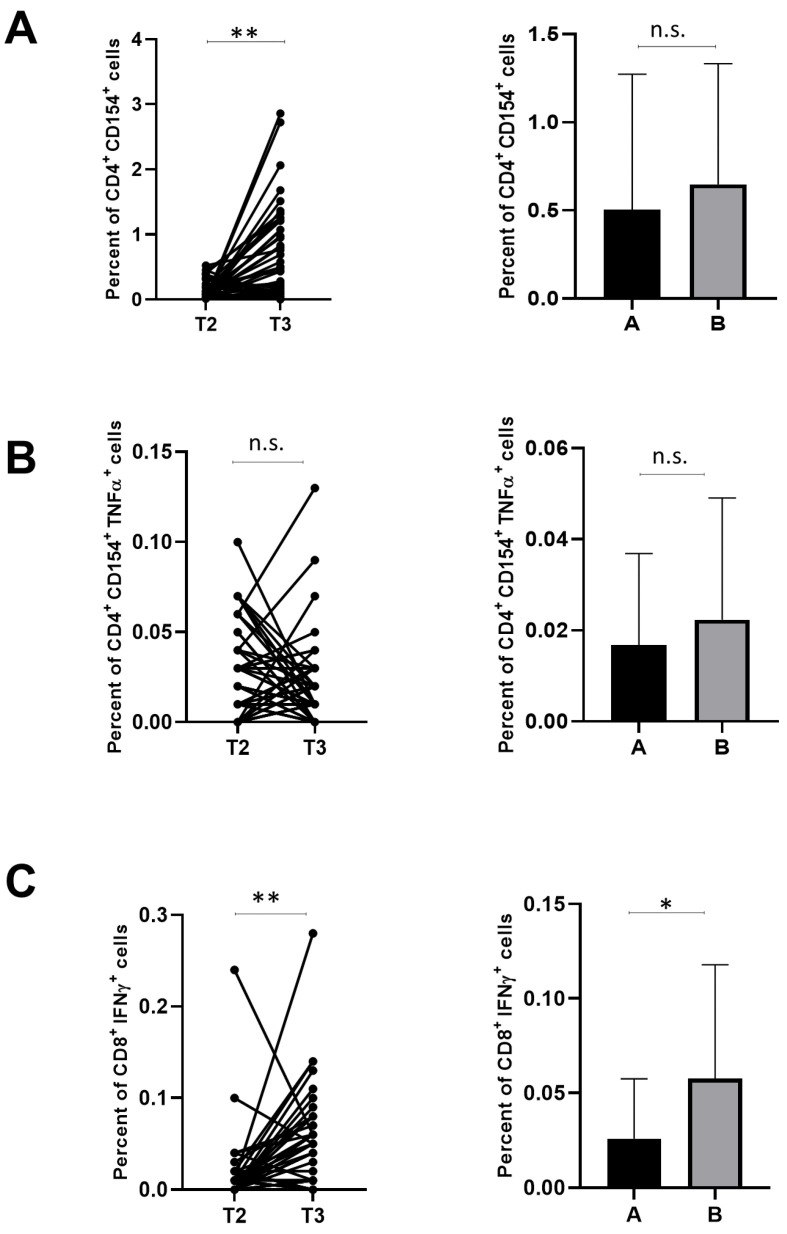
(**A**) CD4^+^ CD154^+^ T cells (percent of total lymphocytes) in 53 family pediatricians (FPs) at T3 compared to T2 (left panel). Differences in the percent of CD4^+^ CD154^+^ cells between patients of group A (n = 20) and group B (n = 33) (right panel). (**B**) CD4^+^ CD154^+^ TNF-α^+^ cells (percent of total lymphocytes) in FP at different time points (left panel). Differences in the percent of CD4^+^ CD154^+^ TNF-α^+^ cells between patients of group A and group B (right panel). (**C**) CD8^+^ IFN-γ^+^ cells (percent of total lymphocytes) in FP at different time points (left panel). Differences in the percent of CD8^+^ IFN-γ^+^ cells between patients of group A and group B; In (**A**–**C**), ** *p* < 0.001; * *p* < 0.05; n.s. not significant. Means and SD are shown. Statistical analysis was performed as reported in the methods.

As shown in Figure 2A, the percentage of CD4^+^ T cells expressing the CD154 activation marker increased significantly in subjects at T3 as compared to T2 (left panel); thus, the booster dose resulted in being essential for the increase of activated CD4^+^ cells. However, we did not observe differences in the percentage of CD4^+^ CD154^+^ cells among patients of group A and group B (right panel). Figure 2B shows that there was no significant difference in the percentage of CD4^+^ CD154^+^ TNF-α^+^ cells between the T2 and T3 time points (left panel) and no significant difference between the A and B groups of patients (right panel). On the other hand, the percentage of CD8^+^ IFN-γ^+^ cells significantly increased in T3 as compared to T2 (Figure 2C, left panels). Furthermore, we observed a significant reduction in the percentage of CD8^+^ IFN-γ^+^ cells in patients of group A when compared to group B (Figure 2C, right panel). Thus, since the percentage of CD8^+^ IFN-γ^+^ cells increased after the booster dose, and it resulted in being higher in FPs unaffected by SARS-CoV-2, these data suggest that a higher number of such cells can be protective against acute infection. Again, we found no differences concerning gender and age.

**Figure 3:**
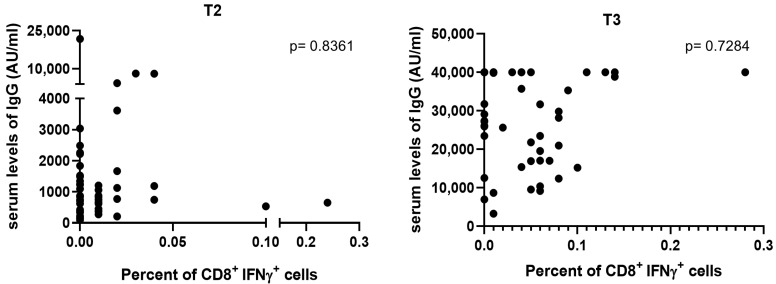
Correlation between serum levels of IgG (AU/mL) targeting the Receptor-Binding Domain (RBD) and percent of CD8^+^ IFN-γ^+^ cells at T2 (**left**) and T3 (**right**). Statistical analysis was performed as reported in the methods.

As shown in Figure 3, no correlation was found between the levels of SARS-CoV-2 S-specific antibodies and CD8^+^ IFN-γ^+^ cells either at T2 or at T3.

**Figure 4:**
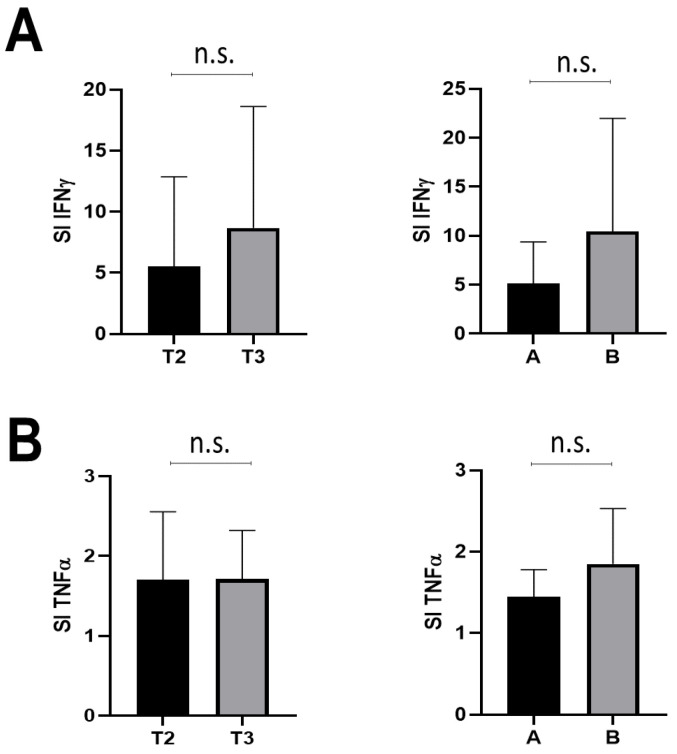
Release (stimulation index, SI) of IFN-γ (panel (**A**)) and TNF-α (panel (**B**)) at T2 and T3 (left panel) in 53 FPs; differences of cytokine release between family pediatricians of group A (n = 20) and group B (n = 33) (right panel). n.s. not significant. Means and SD are shown. Statistical analysis was performed as reported in the methods.

Finally, we assessed the release of TNF-α and IFN-γ by stimulated PBMCs from subjects at either T2 or T3 (Figure 4). No significant difference was recorded for either of the two cytokines after stimulation. However, IFN-γ release increased (although not significantly) in T3 compared with T2 (Figure 4A, left panel). Furthermore, the IFN-γ release was less abundant in group A compared with group B (Figure 4A, right panel), while there were no differences in TNF-α release between time points T2 and T3 or between groups A and B (Figure 4B).

## 4. Discussion

This study aimed to evaluate biomarkers predictive of immunization and protection against the infection by SARS-CoV-2 in a homogeneous cohort of family pediatricians undergoing the three-dose vaccine cycle. Our study demonstrated that all 53 subjects included in the study displayed a humoral response at 1 month from the second dose of the vaccine that significantly declined at 8 months. The booster dose reinforced the humoral response, as assessed one month after the third dose (T3), which resulted in being significantly higher when compared with either T1 (one month) or T2 (eight months post second dose). These data agree with similar studies that reported the decline of the antibody level at 6 months from the vaccine and the relevant increase of the antibody levels after the booster dose [6,22]. However, the antibody level after 1 month from the booster dose was not predictive of immunization toward the acute infection, since we failed to identify any significant difference in the antibody levels between the 20 subjects that developed the infection (confirmed by the molecular swab analysis) and the 33 that did not develop the infection 6 months post the booster dose. Nevertheless, the role of antibody levels against the infection remains unclear. Recent findings support the hypothesis that the measure of mucosal surface IgA should be added to that for circulating IgG. This might result in more protection against infection. Noteworthy, the intramuscular administration of the vaccine is not the optimal way for the induction of the mucosal surface. Further studies are necessary to investigate whether vaccines that induce and trigger a combination of mucosal and systemic immune responses may promote a stronger protection against the infection [23].

The FPs were swabbed about once every 15 days or early in case of symptoms, and all subjects analyzed had no positive swab before the third dose. However, we cannot exclude that in the 15 days, some FPs may have contracted the infection without displaying the symptoms and quickly resulting in a negative at the next swab test. The 20 infected subjects developed the infection in the 6 months following the booster dose, and none required hospitalization or oxygen support, confirming the observations that the booster prevents the severity of disease in a population highly exposed to the risk of being infected, such as FPs, who daily meet a high number of children (and parents) [24,25]. Of notice (see Table 2), during the six months after the third dose, the most frequent virus circulating in Italy was omicron (i.e., B.1.1.529). In countries with documented community transmission, omicron spread significantly faster than the delta variant, with a doubling time of 1.5–3 days. However, this variant was characterized by lower pathogenicity as compared with the previously spreading delta variant (i.e., B.1.617.2). Particularly, omicron was considered the most mutated strain of SARS-CoV-2, considering the large number of mutations as the highly divergent variant. Some of these mutations might be linked to humoral immune escape potential and increased transmissibility. Again, given the high spreading potential, it became the dominant strain on a worldwide scale, contributing to 99.7% of registered sequences from 23 February to 24 March 2022 [26].

Within our cohort, six subjects did not develop a T-cell response (see below) after the vaccine. Notably, all of them experienced the infection during the six months following the booster dose and required more than one week to become negative for the molecular swab test, differently from the other infected subjects.

The evaluation of the T-cell response to the vaccine indicates that the booster dose caused a significant increase in the percent of activated cells (i.e., CD4^+^ CD154^+^ lymphocytes), in agreement with all studies that demonstrated such a response either after the vaccine or after the acute infection [10,27]. However, our data indicate that the percentage of such cells was not predictive of the risk of developing an acute infection in the six months following the booster dose. Furthermore, we demonstrated that the percent of activated T cells expressing TNF-α (i.e., CD4^+^ CD154^+^ TNF-α^+^) did not increase significantly after the booster dose and was not predictive of the risk of developing the acute infection in the six months after the booster dose. This result is further confirmed by the lack of increase in TNF-α release that we observed after the booster dose. Therefore, these findings might suggest that the activated CD4^+^ T cells have a protective role in the prevention of the severe form of the disease [28,29,30]. On the other hand, we observed a trend of increase (although not significant) in IFN-γ release by stimulated cells collected from subjects after the booster dose, with higher levels in those subjects who did not develop the infection in the subsequent six months. Interestingly, we observed a significant increase in T cells expressing IFN-γ (i.e., CD8^+^ IFN-γ^+^) after the booster dose, and this higher frequency is associated with protection against acute infection during the six months after the booster dose [31,32,33]. Moreover, we did not find any correlation between the percentage of CD8^+^ IFN-γ^+^ and the levels of serum antibodies. This finding confirmed that a higher frequency of CD8^+^ IFN-γ^+^ cells may be associated with accelerated viral clearance, and thus, may be considered as a protective factor against acute infection independently from antibody titer [34,35,36,37,38].

In adaptive immunity, CD8^+^ T cells are the principal playmakers in the control of the viral infection by recognizing, attacking, and killing the virus-infected cells and producing effector cytokines. Given the emergence of COVID-19, in the last two years, remarkable progress has been made in understanding CD8^+^ T-cell responses against SARS-CoV-2, and in vivo models have shown that CD8^+^ T cells are able to protect from the development of severe COVID-19 [39,40].

Therefore, these data suggest that the percentage of CD8^+^ IFN-γ^+^ cells following PBMC stimulation can be used as a predictor of protection and provides a clinical indication for subsequent vaccinations.

Among the 53 FPs included in the study, 6 did not develop any T-cell response, i.e., CD8^+^ IFN-γ^+^ cells were absent either after the second or after the booster dose. As previously discussed, all six subjects developed an acute infection during the six months after the booster dose, but did not require hospitalization, indicating that the humoral response (present in all of them) prevented a severe form of the disease. However, in all the subjects, the acute infection lasted longer (i.e., a mean of two weeks before the negative swab test). We speculate that IFN-γ has a main role in viral clearance. In all these 6 subjects, the flow-cytometry panel [41] resulted normal; furthermore, we analyzed a panel of 364 genes related to immunodeficiency [42], excluding any genetic alteration, and we did not find any correlation with age. At the state of the art, we are unable to define the cause of the lack of T-cell response after the vaccine. However, as previously discussed, we cannot exclude that these subjects could have contracted SARS-CoV-2 asymptomatically and became negative quickly during the follow-up window (15 days). A recent study reported that the previous SARS-CoV-2 infection can influence the T-cell response, leading to a decrease of CD8^+^ activation and, hence, to longer viral clearance [43].

We considered a limited number of cases (i.e., 53 subjects) because we preferred to select a homogeneous cohort of highly compliant subjects (i.e., physicians) to obtain full-time monitoring and all clinical data that excluded, in all subjects, the existence of relevant chronic diseases. Moreover, it is still difficult to contextualize all the findings deriving from the pandemic period about the efficacy of the vaccination. More specifically, the global population data in the literature point more toward showing the efficacy of vaccines against severe disease and hospitalization than the efficacy against infection with moderate symptoms. In our case also, about 38% of the subjects resulted in being affected by SARS-CoV-2, and none of them displayed a severe form of the disease. Thus, we know that SARS-CoV-2 has been extremely widespread, infecting 43.9% of the world’s population [44], 44.5% of Italian people, and 44% of residents in Region Campania, according to the data of the Health Ministry in Italy (Italian National Institute of Health). Moreover, we must consider that the FPs were very exposed to the contagion, given their job position.

## 5. Conclusions

In conclusion, our study demonstrates that (i) the BNT162b2-mRNA vaccine against SARS-CoV-2 is followed by the appearance of specific antibodies that significantly increased in serum after the third (booster) dose; however, the serum level of IgG is not predictive of the risk of developing the infection in the six months following the booster dose; (ii) the vaccine induced an increase of activated T cells (i.e., CD4^+^ CD154^+^); (iii) however, the level of CD4^+^ CD154^+^ TNFα^+^ cells, as well as the secretion of TNF-α by activated cells after antigen stimulation, was not modified after the vaccine; (iv) the secretion of IFN-γ by activated cells was increased (although not significantly) after the third dose of vaccine; interestingly, after the booster dose, CD8^+^ IFN-γ^+^ cells significantly increased, and their level was predictive of the risk of developing the infection in the six months following the booster dose, independently of the antibody titer. Furthermore, our study provided additional information on the protection of the three-dose vaccine against severe COVID in a highly exposed population. All such data may impact other virus vaccinations also.

## Figures and Tables

**Table 1:** Study population and sampling time.

Population	
Number of subjects included	53
Median age and range (years)	63 (38–71)
Male/female	17/36
**Vaccination**	
First dose of vaccine	January 2021
Second dose of vaccine	February 2021
Third dose of vaccine (booster dose)	October 2021
**Sampling**	
First sampling (T1)	March 2021
Second sampling (T2)	September 2021
Third sampling (T3)	November 2021
End of the monitoring	May 2022

**Table 2:** Main variants of interest (VOIs) spreading in Italy in the period of the study (January 2021–May 2022) expressed as percentage.

Time Period	Alpha (B.1.1.7)	Beta (B.1.351)	Gamma (P.1)	Delta (B.1.617.2)	Eta (B.1.525)	Kappa (B.167.1)	Omicron (B.1.1.529)	Other
January–March 2021	73.0	0.83	6.0	1.0	1.0	1.2	0	17
September 2021	2.3	0.1	0.4	88.1	0.1	1.2	0.1	8
October 2021	0.1	0.1	0.1	89.6	0	0	0	10
November 2021	0.3	0	0.1	91.4	0	0.9	0	7
December 2021	0.1	0	0.1	86.6	0	0.9	0.1	12
January 2022	0.1	0	0	79	0	0	21	
February 2022	0	0	0	7.1	0	0	71.4	22
March 2022	0	0	0	0.7	0	0.1	90.9	8
April 2022	0	0	0	0.2	0	0	89.7	10
May 2022	0	0	0	0	0	0	93.6	6

## Data Availability

Not applicable.
